# Case report: Combination therapy of envafolimab with endostar for advanced non-small cell lung cancer with low PD-L1 expression

**DOI:** 10.3389/fonc.2024.1437260

**Published:** 2024-11-07

**Authors:** Shuo Wu, Changhong Dong, Chenxi Hu, Kaiyuan Hui, Xiaodong Jiang

**Affiliations:** ^1^ Department of Radiation Oncology, Lianyungang Clinical College of Nanjing Medical University, Lianyungang, China; ^2^ Department of Radiation Oncology, The Affiliated Lianyungang Hospital of Xuzhou Medical University, Lianyungang, China

**Keywords:** non-small cell lung cancer, PD-L1, immune checkpoint inhibitors, anti-angiogenic therapy, envafolimab, endostar, case report

## Abstract

In the management of advanced non-squamous non-small cell lung cancer (NSCLC) without driver gene mutations, the current therapeutic strategies encompass chemotherapy, chemotherapy combined with anti-angiogenic therapy, and chemotherapy combined with immunotherapy. For patients with high programmed death-ligand 1(PD-L1) expression, monotherapy with immune checkpoint inhibitors is a viable option. Recognizing that some patients cannot tolerate or decline chemotherapy, clinical practice has introduced non-chemotherapeutic treatment regimens, which have shown promising results. This article presents a clinical case of advanced NSCLC with low PD-L1 expression and negative driver gene mutations. The patient was treated with a chemotherapy-free regimen combining envafolimab with endostar. After 17 months of follow-up, both the primary tumor and metastatic lesions exhibited significant reduction, and no notable adverse reactions were observed. This case demonstrates the efficacy of envafolimab combined with endostar in the treatment of advanced NSCLC. This regimen enhances treatment safety and patient compliance, potentially offering a novel therapeutic option for patients with advanced NSCLC characterized by low PD-L1 expression and negative driver gene mutations.

## Introduction

Non-Small Cell Lung Cancer (NSCLC) is the most common histological type of lung cancer, accounting for approximately 85% of all lung cancer cases (1). Advances in targeted therapy have improved the prognosis for patients with advanced NSCLC harboring positive driver mutations, yet the benefits for those with negative driver mutations have been limited (2). The first-line treatment for advanced non-squamous NSCLC without driver mutations primarily includes chemotherapy, chemotherapy combined with anti-angiogenic agents, and chemotherapy combined with immunotherapy. For patients with high programmed Death-Ligand 1 (PD-L1) expression, monotherapy with immunotherapy is an option (3).

Envafolimab is the world’s first PD-L1 inhibitor administered via subcutaneous injection and the first immunotherapy drug in China approved for a broad range of solid tumor indications, specifically for adult patients with unresectable or metastatic MSI-H/dMMR solid tumors (4). Endostar is an endogenous multi-target anti-angiogenic agent that exerts its anti-angiogenic effects by blocking the tyrosine phosphorylation of the KDR/Flk-1 receptor induced by vascular endothelial growth factor (VEGF). Additionally, it inhibits the migration and invasion of vascular endothelial cells by suppressing integrin and matrix metalloproteinase 2, thereby inhibiting tumor growth and metastasis (5). As a classic anti-angiogenic drug, Endostar has demonstrated satisfactory efficacy and good safety in both squamous and non-squamous NSCLC patients, and it is recommended as a treatment option for locally advanced and advanced NSCLC. It can also be used in combination with chemotherapy as a first-line treatment for patients with advanced NSCLC without driver mutations (6).

Immune checkpoint inhibitors (ICIs) and anti-angiogenic drugs complement each other synergistically. ICIs promote the normalization of tumor vasculature by activating T cells to secrete cytokines, while anti-angiogenic drugs enhance the formation of normal vasculature while improving the immunosuppressive microenvironment. The combination of these two therapies maximizes the antitumor effect (7). Previous studies have indicated the effectiveness of combining immunotherapy with anti-angiogenic treatment in patients with low PD-L1 expression, but specific efficacy data have rarely been reported. This article presents a case of a patient with advanced non-small cell lung cancer (NSCLC) characterized by low PD-L1 expression and negative driver mutations who received first-line treatment with a combination of envafolimab and endostar. The safety and efficacy of this treatment regimen are discussed.

## Case presentation

**Figure 1:**
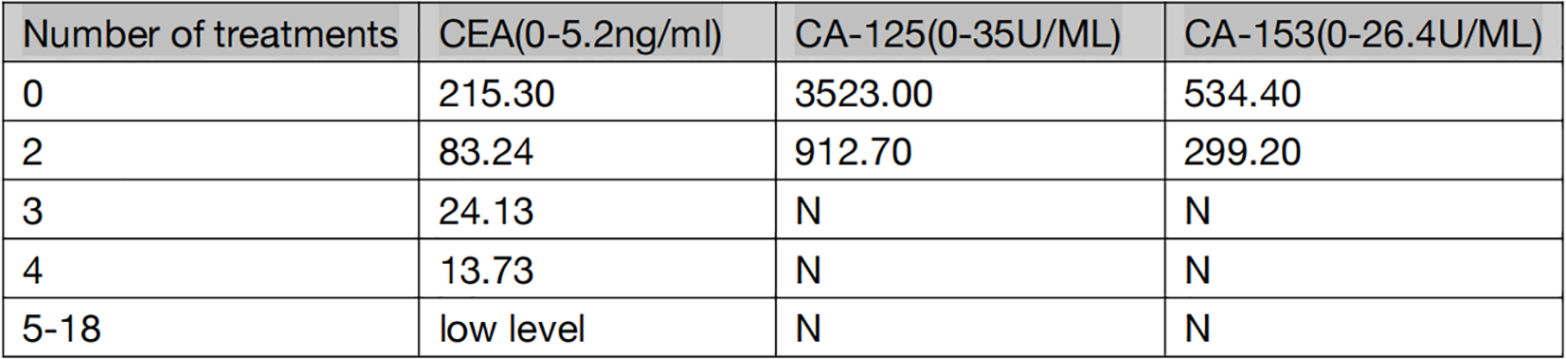
Changes of tumor indexes after treatment. N=normal.

**Figure 2:**
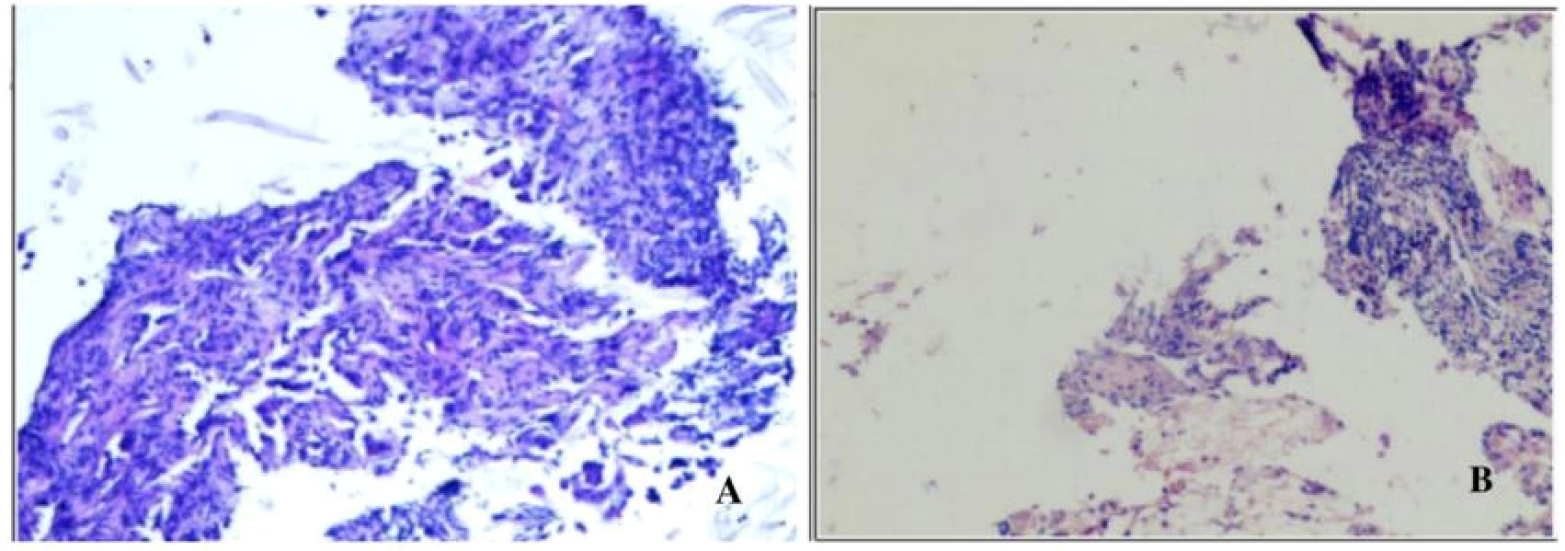
**(A)** Pathological findings of cervical lymph node fine-needle aspiration biopsy showing a tendency towards metastatic adenocarcinoma stained with Hematoxylin and Eosin (HEx200). **(B)** Tissue biopsy from the vertebral lesion demonstrating adenocarcinoma stained with Hematoxylin and Eosin (HEx200).

The patient is a 59-year-old male with no history of smoking or alcohol consumption. On September 13, 2022, the patient presented to the spine surgery department with complaints of neck pain and restricted movement. The Eastern Cooperative Oncology Group (ECOG) performance status (PS) was assessed as 1. A computed tomography (CT) scan of the neck and chest on September 14, 2022, revealed a right lower lobe lung mass suggestive of possible lung cancer. Additionally, there were findings indicative of possible metastatic lesions, including involvement of the left occipital bone, C2 vertebral body, left T2 appendix, T7 vertebral body, right scapula, left ilium, and pubic bone with osteolytic changes. Enlarged mediastinal and right axillary lymph nodes were also observed. Ultrasound examination of the supraclavicular lymph nodes showed multiple hypoechoic nodules on the left side, suggestive of lymph node metastasis. No significant abnormal lymph nodes were detected in the right side of the neck and supraclavicular fossa.Blood tumor markers revealed elevated levels of CA-125, CA-153, and CEA compared to normal ranges (specific values are presented in **Figure 1**). On September 20, 2022, the patient underwent a cervical lymph node fine-needle biopsy, with pathological findings suggestive of metastatic adenocarcinoma (**Figure 2A**). Immunohistochemistry results showed: CK (3+), CK5/6 (-), P40 (-), NapsinA (-), TTF-1 (-), CD56 (-), Syn (-), CK7 (3+). On September 23, 2022, the patient underwent posterior cervical fusion surgery in the spine surgery department. Postoperative histopathological analysis of vertebral lesion tissue indicated adenocarcinoma (**Figure 2B**) with immunohistochemistry results as follows: CK7 (focally 3+), TTF-1 (-), NapsinA (-), CK8/18 (focally 3+), CK5/6 (-), CK20 (-), CDX-2 (-), CD56 (-), PSA (-). The final diagnosis for the patient was stage IV right lung adenocarcinoma (cT1N3M1).

**Figure 3:**
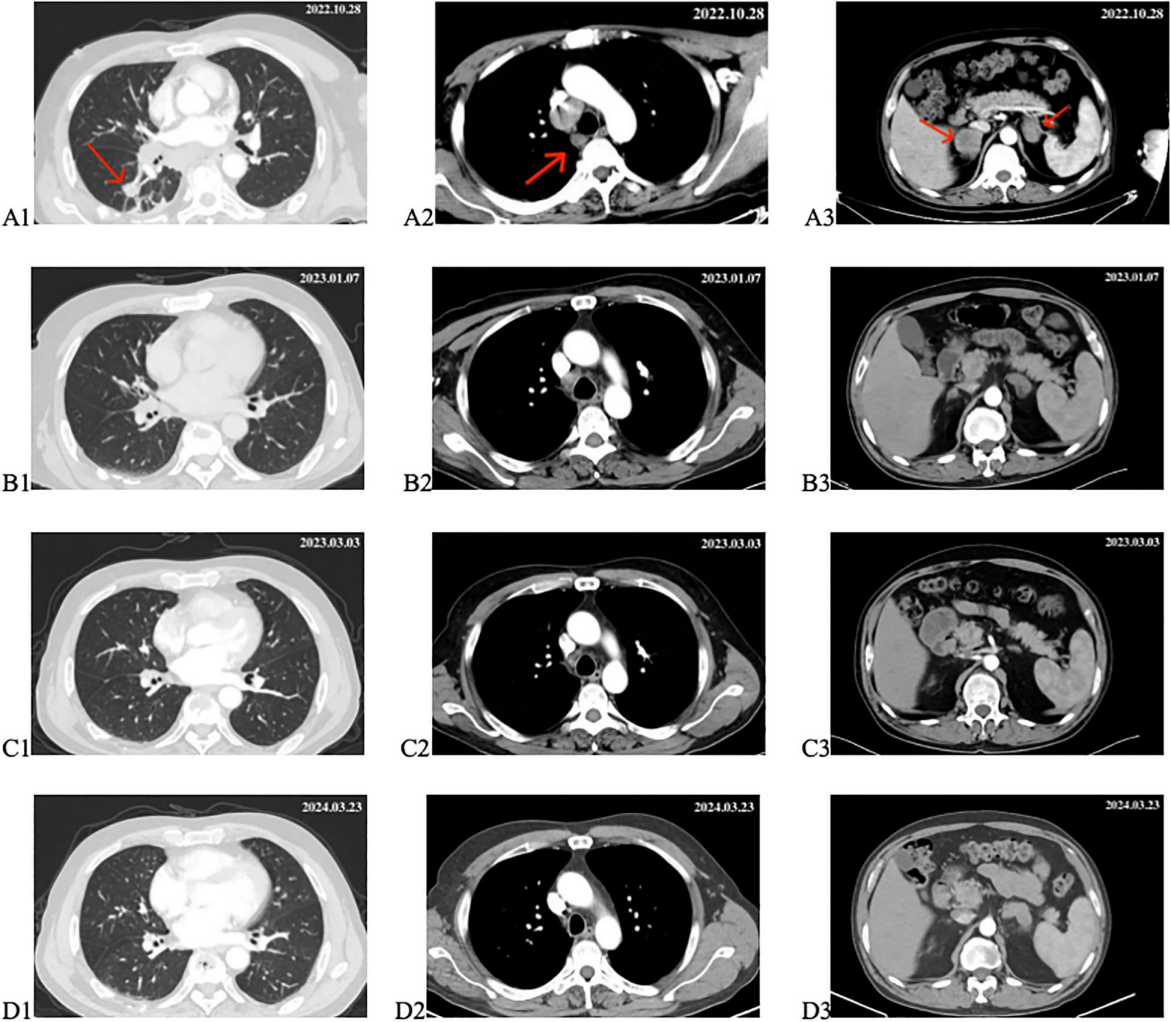
Changes in target lesion during treatment. **(A1–A3)** CT scan before treatment. **(B1–B3)** CT scan after 2 cycles of treatment. **(C1–C3)** CT scan after 4 cycles of treatment. **(D1–D3)** CT scan after 18 months of treatment. The red arrows mark the target lesions.

On October 28, 2022, the patient visited the oncology department. A repeat chest and abdominal CT scan on the same day showed a lesion in the right lung measuring approximately 16*13mm; multiple metastatic lymph nodes were observed in the right hilar, mediastinum, and right axilla, with bilateral adrenal metastases, and a small amount of pleural effusion on the right side (**Figures 3A1–A3**). Driver gene testing for epidermal growth factor receptor, anaplastic lymphoma kinase, and C-ROS oncogene 1 in the biopsy tissue revealed no mutations, and the PD-L1 testing result was tumor proportion score (TPS) of 1%. According to the Chinese Society of Clinical Oncology guidelines, the patient was recommended chemotherapy, ICIs combined with chemotherapy, or anti-angiogenic therapy combined with chemotherapy. However, the patient refused chemotherapy. After thorough discussion with the patient and family members, the patient opted for ICIs combined with anti-angiogenic therapy. The specific regimen included subcutaneous injections of envafolimab at 300mg per dose combined with intravenous infusion of endostar at 210mg over 72 hours, administered once every 3 weeks. Following comprehensive pre-treatment evaluations and exclusion of any contraindications, the patient commenced treatment on November 1, 2022.

From November 1, 2022, to December 2, 2022, the patient underwent two cycles of treatment with envafolimab in combination with endostar. After these two cycles, the patient was admitted for a follow-up examination. A chest and abdominal CT scan (January 7, 2023) revealed a reduction in the size of the right lung lesion, decreased size of mediastinal lymph nodes, disappearance of axillary lymph nodes, decreased size of adrenal metastases on both sides, and resolution of pleural effusion. According to RECIST 1.1 criteria, the treatment response was evaluated as partial remission (PR) (**Figures 3B1–B3**). The blood tumor markers CA-125, CA-153, and CEA exhibited significant decreases (**Figure 1**). From January 8, 2023, to February 3, 2023, the patient underwent the third and fourth cycles of anti-tumor treatment. After three courses of treatment, CA-125 and CA-153 levels decreased to within the normal range (**Figure 1**). Following four courses of treatment, CEA levels showed a marked decline and subsequently remained at a low level (**Figure 1**). Upon CT re-evaluation on March 3, 2023, the results indicated: the right lung lesion had nearly disappeared, the mediastinal lymph nodes had shrunk, the axillary lymph nodes had vanished, the bilateral adrenal metastases had reduced, and the bone metastatic lesions showed increased bone density (**Figures 3C1–C3**). The tumor regression was evident, and the efficacy was assessed as Partial Response (PR). From March 3, 2023, to February 23, 2024, the patient underwent cycles 5-18 of treatment, maintaining a PR status. On March 23, 2024, a follow-up CT scan revealed that the bilateral adrenal metastases had enlarged compared to previous scans, suggesting disease progression (**Figures 3D1–D3**). Consequently, the treatment regimen was discontinued, with the patient’s progression-free survival (PFS) recorded as 16.7 months. It is noteworthy that during the treatment period, the patient experienced only mild gastrointestinal reactions.

## Discussion

In this case, the patient was diagnosed with stage IV lung adenocarcinoma (TNM staging), with negative driver gene test results and a very low PD-L1 expression level (TPS=1%). As the patient was unwilling to undergo chemotherapy, a treatment regimen combining Endostar (endostatin) with Envafolimab was administered. After 18 cycles of treatment, the patient’s lung lesions disappeared, achieving complete remission (CR). The bone metastatic lesions exhibited osteogenic changes, the metastatic lymph nodes shrank, and the pleural effusion resolved. The duration of response lasted for 16.7 months, indicating sustained efficacy. It is noteworthy that the patient did not experience any severe treatment-related adverse reactions during the course of therapy. This suggests that this treatment regimen was both safe and effective for this patient.

For lung cancer patients without driver gene mutations, the combination of PD-1/PD-L1 inhibitors with chemotherapy has shown promising efficacy (8). This is particularly evident in patients with high PD-L1 expression. In recent years, several studies have begun to investigate the efficacy of immunotherapy in patients with low PD-L1 expression. For instance, the KEYNOTE-407 clinical trial demonstrated that the combination of pembrolizumab with chemotherapy can improve the 5-year overall survival (OS) rate in patients with stage IV squamous NSCLC, regardless of PD-L1 expression levels (9). Although subgroup analyses indicated that the primary beneficiaries were patients with PD-L1 ≥ 50%, extended cohort data from KEYNOTE-189 and KEYNOTE-407 suggested that pembrolizumab plus chemotherapy also has the potential to improve OS, objective response rate (ORR), and PFS in patients with PD-L1 < 1% (10). The IMpower150 clinical trial randomly assigned patients with stage IV or recurrent metastatic non-squamous NSCLC who had not received prior chemotherapy to one of three treatment arms: atezolizumab plus carboplatin and paclitaxel (ACP group), atezolizumab plus bevacizumab, carboplatin, and paclitaxel (ABCP group), or bevacizumab plus carboplatin and paclitaxel (BCP group) in a 1:1:1 ratio. The results showed significant improvements in PFS and OS in the ABCP group, which added atezolizumab to the BCP regimen. The IMpower150 study also revealed that, among patients with low or negative PD-L1 expression, the 12-month PFS rate was twice as high in the ABCP group compared to the BCP group (36.5% vs. 18.0%), possibly due to bevacizumab enhancing the efficacy of atezolizumab (11). While ICIs combined with platinum-based doublet chemotherapy represent the standard first-line treatment for advanced NSCLC driven by negative mutations, the associated side effects can be substantial, leading to poor patient adherence (12). For patients with poor physical condition or those who cannot tolerate chemotherapy, this treatment approach may not be suitable (13). Therefore, there is an urgent need for a therapeutic regimen that is both efficacious and well-tolerated. The non-chemotherapeutic combination of Endostar and Envafolimab may offer a novel treatment strategy.

Blood vessels play a pivotal role in tumor growth, infiltration, and metastasis. Anti-angiogenic drugs target various receptors such as vascular endothelial growth factor receptor, platelet-derived growth factor receptor and fibroblast growth factor receptor (VEGFR, PDGFR, and FGFR), along with their downstream signaling pathways, to impede tumor angiogenesis. Despite the efficacy of single-agent anti-angiogenic therapy, resistance can arise, limiting its effectiveness (14). ICIs function by revitalizing the body’s anti-tumor immune response, leading to cancer cell destruction. In comparison to traditional cytotoxic chemotherapy, ICIs offer higher response rates, extended overall survival, and improved tolerability (15). Nevertheless, single-agent immunotherapy typically yields a response rate of only 20%-30%. Consequently, combination therapy has emerged as a critical strategy to enhance cancer treatment outcomes (16). Phase II clinical trials have demonstrated that the combination regimen of apatinib and camrelizumab exhibits encouraging anti-tumor activity with manageable toxicity in patients with advanced non-squamous NSCLC who have undergone prior chemotherapy (17). Another study, the NCT0362852 clinical trial, involved treatment-naïve patients with unresectable stage IIIB/C or IV non-small cell lung cancer without driver gene mutations. These individuals received combined treatment involving sintilimab and anlotinib every three weeks, resulting in a 100% disease control rate and a median PFS of 15 months (18). Therefore, the combination therapy of immune checkpoint inhibitors (ICIs) with anti-angiogenic agents has demonstrated promising clinical benefits and good safety in patients with advanced non-small cell lung cancer (NSCLC). For elderly and physically frail cancer patients, the safety and adherence of anti-tumor treatments are particularly important. Compared to other anti-angiogenic drugs, Endostar has a broader range of applicability, and its continuous intravenous infusion is associated with fewer side effects and higher safety (5). Envafolimab, as the first subcutaneously injected PD-L1 immune checkpoint inhibitor, has emerged as a new treatment option for various types of cancer due to its remarkable efficacy and minimal side effects (19). The combination of these two therapies may yield unexpected results. In this particular case, the patient’s progression-free survival (PFS) was 1.7 months longer than the median PFS observed in the NCT0362852 clinical trial, although this comparison did not reach statistical significance. Nevertheless, it highlights the feasibility of this treatment regimen. Therefore, more research is needed to further validate the efficacy and advantages of this combination therapy. This treatment approach, combining Endostar with Envafolimab, could be promoted among similar elderly patients or those who decline chemotherapy due to concerns about toxic side effects.

Furthermore, the patient in this case only experienced mild gastrointestinal reactions during treatment, and symptoms were alleviated after symptomatic treatment. In larger population studies, adverse reactions in patients should be given additional attention.

Lastly, considering economic reasons or other factors, further research could be conducted on the combination of Endostar and Envafolimab with other immune inhibitors or anti-angiogenic drugs, respectively. The efficacy of these combinations could then be compared to the combination of Endostar and Envafolimab, with the aim of exploring more diverse and appropriate treatment options for patients.

In this case, the patient achieved favorable treatment outcomes and good safety with a chemotherapy-free combination therapy of Endostar and Envafolimab, which also improved patient compliance. The application of this regimen in this case provides a new treatment approach for similar patients and has the potential to transform the treatment paradigm for non-small cell lung cancer (NSCLC) patients who are intolerant to chemotherapy. However, further clinical studies are still needed to validate these findings.

## Data Availability

The original contributions presented in the study are included in the article/supplementary material. Further inquiries can be directed to the corresponding authors.
